# A Comprehensive Study of Uterine Muscle Activity During the Third Trimester: Comparison of Singleton and Multiple Gestations

**DOI:** 10.1109/JTEHM.2025.3637293

**Published:** 2025-11-26

**Authors:** Yu Meng, Javier Garcia-Casado, Gema Prats-Boluda, Jose Luis Martinez-de-Juan, Carmen Padilla Prieto, Rogelio Monfort-Ortiz, Vicente José Diago-Almela, Dongmei Hao, Guangfei Li, Yiyao Ye-Lin

**Affiliations:** Centro de Investigación e Innovación en BioingenieríaUniversidad Politécnica de Valencia Valencia 46022 Spain; Servicio de ObstetriciaHospital Universitario y Politécnico de La Fe Valencia 46026 Spain; Beijing International Science and Technology Cooperation Base for Intelligent Physiological Measurement and Clinical TransformationDepartment of Biomedical Engineering, College of Chemistry and Life ScienceBeijing University of Technology 12496 Beijing 100124 China; BJUT-UPV Joint Research Laboratory in Biomedical Engineering Beijing 100124 China

**Keywords:** Electrohysterogram (EHG), generalized additive models, multiple gestation, preterm birth, singleton gestation, uterine contraction monitoring

## Abstract

Objective: Electrohysterography (EHG) has been shown to provide valuable information for assessing preterm birth risk. However, few studies have focused on multiple gestations (MG), a well-known risk factor for preterm birth. This study aimed to comprehensively characterize and compare uterine EHG signals between singleton (SG) and MG pregnancies during the third trimester. Method: This prospective cohort study analyzed 383 EHG recordings from 61 SG and 92 MG women during the third trimester. A whole-window approach was used to extract four key EHG features: peak-to-peak amplitude (PPA), Kurtosis of the Hilbert Envelope (KHE), median frequency (MDF) and sample entropy (SampEn). Generalized additive models (GAM) were applied to evaluate temporal trends across gestational age (GA) and gestation type (SG and MG). Results: In SG pregnancies, PPA and KHE progressively increased, with a significant rise in KHE at labour. MDF remained stable until labour, while SampEn gradually declined, especially at term. MG pregnancies showed similar but less pronounced trends: MG exhibited a notably earlier activation of uterine activity than SG before 32 weeks of gestation (WoG), and a slowing-down electrophysiological progression beyond 32 WoG, resulting in similar characteristics with no significant differences. Conclusion: These findings provide electrophysiological evidence suggesting that MG pregnancies may enter a labour-preparatory state earlier, potentially increasing the PTB risk, while the later convergence of EHG features may indicate compensatory mechanisms to delay labour. This work integrates EHG signal analysis with clinical obstetric care, offering valuable insights for clinical management and early PTB risk assessment in MG pregnancies.

## I. Introduction

Preterm birth (PTB), defined as delivery before 37 weeks of gestation (WoG), remains a pressing global health issue, with an incidence exceeding 10% as of 2010 [1]. It is a leading cause of neonatal morbidity and mortality, with long-term implications for child development and socioeconomic outcomes [2]. Various tools have been proposed for PTB risk assessment, including obstetric history and clinical examination-based risk scoring systems, but their predictive performance is limited by confounding factors such as ethnicity and lifestyle [3]. Biomarker-based methods, such as measurement of interleukin-6 (IL-6) in amniotic fluid and fetal fibronectin in cervicovaginal secretions, have shown promise but suffer from variable accuracy and an uncertain correlation with delivery timing [4], [5]. Transvaginal ultrasound is currently regarded as a reliable tool for evaluating the PTB risk, although variations in cervical length thresholds across populations limit its general applicability [6]. Tocodynamometry (TOCO) is a conventional method used to monitor uterine contractions. In addition to issues with transducer repositioning, TOCO shows poor performance in obese patients, as its sensitivity significantly decreases with increasing maternal body mass index (BMI) and abdominal circumference, due to subcutaneous adipose tissue attenuates the mechanical deformation generated by uterine contractions [7].

Uterine electrohysterogram (EHG) has emerged as a low-cost, non-invasive tool for monitoring uterine activity by dynamically capturing bioelectrical signals from the myometrium [8]. Growing evidence indicates that EHG outperforms TOCO in monitoring uterine dynamics in obese patients [7], [9]. EHG signals comprise both slow and fast wave components, although the recent focus has shifted toward fast waves (FW) due to difficulties in interpreting the slow waves [8]. FW can be further categorized into fast waves low (FWL), with peak frequencies between 0.13 and 0.26 Hz, and fast waves high (FWH), with peak frequencies between 0.34 and 4 Hz, which are associated with signal propagation and uterine cellular excitability, respectively [9]. Previous studies have demonstrated that EHG parameters, such as signal amplitude [10], peak frequency [11], and nonlinear parameters like sample entropy (SampEn) [12] can effectively differentiate between term and preterm labour.

Although numerous computational parameters derived from EHG signals have been proposed to characterize the uterine electrophysiological condition, their value across different stages of pregnancy remains under debate. While some studies reported significant increases in amplitude-related features such as Root Mean Square (RMS) and peak-to-peak amplitude prior to the onset of labour [13], others found this parameter made no reliable distinction between term and preterm deliveries [12]. Frequency-domain features also lack consistent descriptions across studies, e.g. Verdenik et al. reported a slight decrease in median frequency with advancing gestational age (GA) [14], whereas other findings suggested that an increase in frequency may indicate impending labour [15]. Nonlinear measures like SampEn have also obtained controversial results. In the uterine corpus, SampEn was found to decrease continuously after the onset of active labour, while in the cervix it showed a sudden drop prior to active labour and remained stable thereafter, highlighting that spatial dependency makes its interpretation more challenging [16]. These inconsistencies underline the need for further research into the dynamic evolution of EHG parameters, particularly during the third trimester and the transition to labour and understanding these patterns may serve as the cornerstone of the clinical utility of EHG in predicting preterm birth.

Notably, most PTB studies have focused on singleton gestations (SG), whereas multiple gestations (MG) —defined here as twin gestations—have a significantly higher PTB risk, reaching up to 60% in twin pregnancies compared with approximately 10% in SG [1]. Traditional PTB prediction tools, including cervical length and fetal fibronectin testing, are less effective or inconsistent in MG [17]. These findings suggest distinct physiological and immunological mechanisms in MG that drive myometrial adaptations during the third trimester, forming the basis of our hypothesis [18]. Emerging evidences suggest that excessive uterine distension in MG triggers inflammatory responses and increases uterine excitability through stretch-activated ion channels [19], potentially leading to an altered signaling pathway. Indeed, MG has been reported to exhibit accelerated electrophysiological changes toward labour for early third trimester (from 28 to 32 weeks of gestation), resulting in an incomplete or prematurely activated EHG pattern for MG [20]. Assessing electrophysiological differences between singleton and multiple gestations throughout the third trimester is crucial for advancing our understanding of the physiological mechanisms underlying preterm birth and for improving the accuracy of preterm birth risk prediction. The present study therefore aimed to systematically characterize and compare the dynamic evolution of uterine electrical activity between SG and MG pregnancies during the third trimester. By providing detailed electrophysiological profiles of uterine activity, this work advances understanding of the physiological mechanisms in myometrial activation and may also inform future strategies for early identification of PTB risk, particularly for MG pregnancies with an inherently elevated risk.

## II. Data and Methods

### A. Study Population

This study was a prospective cohort study of pregnant women undergoing routine prenatal check-ups at the Polytechnic and University Hospital La Fe (Valencia, Spain). The exclusion criteria included conditions such as fetal compromise, severe maternal disease or obstetric complications, which could introduce a bias [20]. The study was approved by the Ethics Committee of Hospital Universitario y Politécnico La Fe, Valencia, Spain, under register number 2025-0191-1. All the participants were informed of the study objectives and data collection procedures, and written informed consent was obtained from each one. The collected clinical information included GA at the time of EHG recording, maternal age (chronological age in years), parity status, history of cesarean section, and history of abortion.

The study population comprised 147 and 236 EHG recordings collected from 61 SG and 92 MG women, respectively, whose demographic and obstetric characteristics are shown in Table 1. In this study, we only included twin gestations, no triplet or higher-order multiple gestations were included. Due to the low prevalence of MG in natural conception [21], we prioritized the recruitment of MG patients among women undergoing assisted reproductive treatment, in whom the prevalence can reach approximately 30–35% [22]. To address the significant GA difference between the groups in the initial dataset, a stratified dynamic sampling method was applied to minimize its impact on the analysis. This method involved segmenting the GA into fixed-width intervals, grouping the data within each interval, and applying a dynamic downsampling strategy to reduce the size of the larger group while retaining as much information as possible. This approach was based on dynamic sampling techniques originally developed for data assimilation in environmental models, which have demonstrated strong performance in maintaining distribution consistency while controlling the computational cost [23]. The resulting GA-balanced dataset included 110 and 117 EHG recordings from 53 SG and 63 MG women, respectively (see Table 1).

**TABLE 1:** Demographic and Obstetric Characteristics of Singleton and Multiple Pregnancies of Original and GA-Balanced Dataset After Stratified Dynamic Sampling

	Original dataset			GA-Balanced dataset		
Variable	SG (N= 61)	MG (N= 92)	P-value	SG (N= 53)	MG (N= 63)	P-value
Number of Signal Records	147	236	–	110	117	–
Maternal Age (years), $\mu $± $\sigma $	33.6 ± 4.5	35.0 ± 4.3	0.042	33.9 ± 4.5	35.0 ± 4.4	0.203
GA at EHG (days), $\mu $± $\sigma $	213.4 ± 18.7	235.5 ± 18.9	<0.001	218.9 ± 18.3	223.6 ± 19.5	0.064
GA at delivery (days), $\mu $± $\sigma $	39.3 ± 1.7	35.8 ± 1.4	<0.001	39.3 ± 1.8	35.7 ± 1.5	<0.001
Parity (Multiparous/Nulliparous) (N/total)	22/61	17/92	0.022	22/53	17/63	0.099
Cesarean Section History (N/total)	7/61	4/92	0.094	7/53	4/63	0.209
Abortion History (N/total)	16/61	19/92	0.693	16/53	19/63	0.997

We also included two additional groups of singleton pregnancies to fully describe the uterine electrophysiological changes from the third trimester of pregnancy until delivery: 17 term no labour (TNL) women at 39 WoG and 10 women in the active phase of labour (APL) with GA >40 WoG, with one recording per woman.

### B. EHG Signal Acquisition

Each participant underwent at least 30 minutes of EHG recording and was instructed to remain as still as possible to minimize motion artifacts. For each recording, the abdominal skin was exfoliated with Nuprep gel (Weaver and Company, Aurora, CO, USA) and then cleaned with isopropyl alcohol to reduce skin-electrode impedance.

**FIGURE 1.**
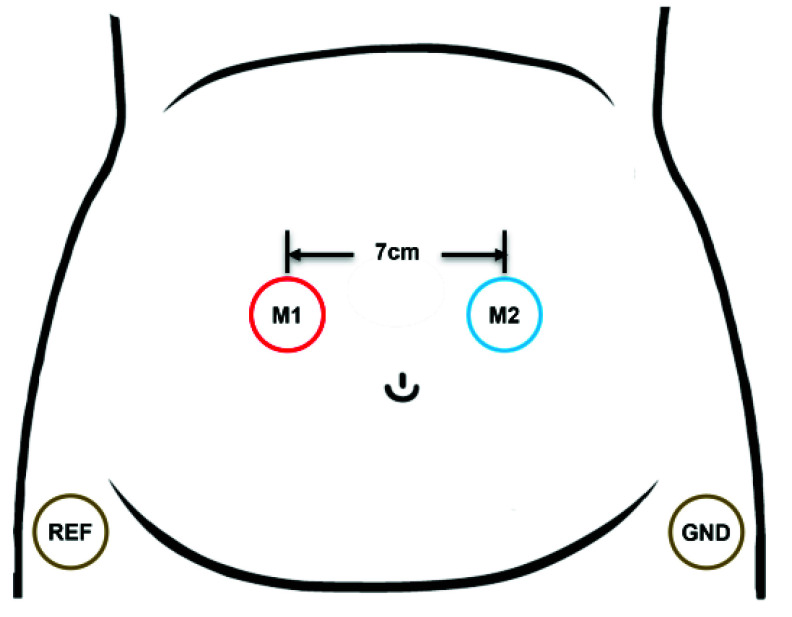
Electrode placement for uterine myoelectrical recording.

EHG signals were recorded following the electrode placement shown in Fig. 1. Two disposable Ag/AgCl electrodes (Red Dot 2660–5, 3M, St. Paul, MN, USA) were symmetrically positioned along the midline between the symphysis pubis and the uterine fundus, corresponding to the region of greatest myometrial muscle fibre density [24]. This configuration is equivalent to the E3 electrode pair of the public TPEHG database, which has been validated for reliable EHG acquisition as early as 26 weeks of gestation and for discriminating between preterm and term deliveries [12], [25], [26]. An inter-electrode distance of 7 cm was chosen to ensure adequate signal amplitude while minimizing the blurring effects of volume conduction, which is particularly relevant during the early third trimester when EHG amplitudes are weak [27]. The reference and ground electrodes were placed on the left and right iliac crests, respectively, to provide stable bioelectric potentials [28].

To streamline data acquisition, all signals were recorded using a custom wireless recording module with the following specifications: gain of 2059 V/V, a bandwidth of 0.1–150 Hz, and a sampling rate of 500 Hz, digitized with a 24-bit analog-to-digital converter [29].

The raw data was preprocessed to keep the main components in 0.1-4 Hz to retain the physiologically meaningful fast-wave components of the EHG while filtering out low-frequency baseline drift and high-frequency noise, and downsampled to 20 Hz [30]. To ensure data quality, we visually discarded the motion artifacts and respiration interference from EHG recording using the criteria proposed by Felix [31], being the percentage of data rejection about 20.6%. Two experts conducted a double-blind review to minimize subjective bias.

### C. Whole-Window Analysis for EHG Feature Extraction

Due to the low amplitude of uterine contractions during pregnancy, which may not be clearly distinguishable from basal activity, we employed a whole-window analysis approach instead of the conventional EHG burst analysis. Growing evidence has demonstrated the feasibility of whole-window analysis in characterizing uterine EHG for preterm birth prediction, which even outperforms EHG burst analysis [8], [32]. In this study, we used a 120-second sliding window with a 50% overlap, which has been shown to provide an optimal balance between temporal resolution and statistical robustness [32].

Four widely used features were extracted to characterize time, frequency and nonlinear domains. In the 0.1–4 Hz band, Peak-to-Peak Amplitude (PPA) and Kurtosis of the Hilbert Envelope (KHE) were computed to reflect contraction intensity and signal impulsiveness, respectively [32], [33]. Median Frequency (MDF) was calculated in the 0.2–1 Hz range to reduce cardiac interference while reflecting cellular excitability [34]. For nonlinear characterization, SampEn was computed in the 0.34–4 Hz range (FWH) using a template length of 2 and tolerance of 0.2 (m = 2 and $r = 0.2$), to quantify signal complexity which has previously been shown to be effective in distinguishing term and preterm deliveries [12], [33]. All features were computed from the original filtered signals without normalization [35], and the median value across all windows was used to represent each recording. These measures capture complementary information of uterine electrical activity—amplitude, waveform morphology, frequency content, and dynamical regularity—which collectively reflect the population activation level and underlying excitability of the myometrium, both physiologically and clinically relevant for labor prediction.

### D. Statistical Analysis

Since EHG features during the third trimester exhibit nonlinear temporal dynamics, we used generalized additive models (GAM) for smoothed, non-parametric curve fitting to better capture nonlinearity [36]. GAM is a flexible regression model that captures nonlinear relationships using smooth functions, making it ideal for analyzing complex EHG feature trends over time [37]. Since EHG features may have nonlinear relationships with GA, we used the Spearman’s rank correlation coefficient to gauge their interaction.

Clinically, 32 WoG represents a critical threshold for fetal viability and neonatal outcomes, with preterm births before 32 WoG associated with significantly higher risks of complications [38], [39], obstetric classifications also define very preterm birth as <32 WoG [40]. Consequently, we stratified the routine prenatal check-up population, including both SG and MG into two subgroups: early third trimester (Early3T, GA <32 WoG) and late third trimester (Late3T, $32\le $ GA <37 WoG). Table 2 provides the number of women and EHG recordings across six groups defined by gestational stage and pregnancy type. The Wilcoxon rank-sum test ( $\alpha = 0.05$) was used to statistically assess (1) differences between SG and MG within the same GA group, and (2) differences across different GA groups within the same pregnancy type (SG or MG).

**TABLE 2:** Number of EHG Recordings (and Women) Stratified by GA for SG and MG

Group	Early3T	Late3T	TNL	APL
	(26,32] WoG	(32,37) WoG	39 WoG	[40, 41] WoG
SG	83 (50)	27 (20)	17 (17)	10 (10)
MG	68 (39)	49 (37)	NA	NA

## III. Results

**FIGURE 2.**
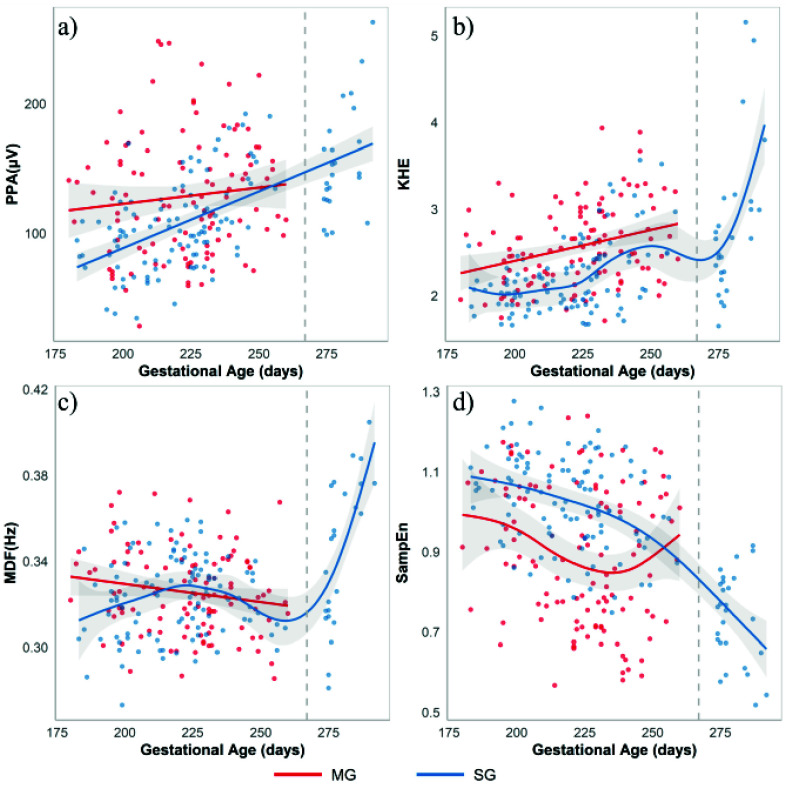
Gestational age-dependent trends of EHG features in SG (blue) and MG (red) pregnancies. Each dot represents one EHG recording. Solid lines indicate the trends estimated by GAM, with shaded areas showing the 95% confidence intervals. The vertical dashed line at 37 WoG marks the boundary between data collected during routine check-ups and those from full-term (TNL and APL) cases.

Fig. 2 shows the GA-dependent trends of EHG Parameters for SG and MG. Table 3 summarizes the Spearman correlation coefficients between GA and EHG parameters for both SG and MG pregnancies.

**TABLE 3:** Spearman Correlation Coefficient ( $\rho $) and p-Values Between EHG Features and GA in Singleton and Multiple Pregnancies

Features	MG		SG	
	$\rho $	P Value	$\rho $	P Value
PPA	0.133	0.176	0.604	<0.001
KHE	0.303	0.001	0.509	<0.001
MDF	-0.149	0.127	0.239	0.007
SampEn	-0.179	0.054	-0.608	<0.001

As GA increases, PPA exhibits a gradual and upward trend in both SG and MG pregnancies, obtaining a strong ( $\rho = 0.604$) and statistically significant correlation (p-value<0.001) in SG. By contrast, no significant relationship between PPA and GA was found in MG.

KHE also exhibited a nonlinear increasing trend with GA in both SG and MG pregnancies. This trend is particularly pronounced beyond 37 WoG in SG, where a sharp rise in KHE is observed as labour approaches. Both SG and MG showed a statistically significant correlation with GA, this correlation being more pronounced in SG ( $\rho = 0.509$ in SG vs. $\rho = 0.303$ in MG). It should be noted, however, that the correlation in SG was calculated over a broader GA range, including the term and active phase of labour recordings, whereas the MG analysis was restricted to data before 37 WoG, which may partially account for the stronger correlation observed in SG.

Although MDF in both SG and MG pregnancies remained relatively stable during routine prenatal check-ups (before 37 WoG), a notable surge was seen in SG pregnancies near the time of delivery, which contributed to a significant positive correlation between MDF and GA in this group ( $\rho = 0.239$, p-value = 0.007).

SampEn showed a nonlinear downward trend as pregnancy progresses for SG, obtaining a strong and statistically significant correlation ( $\rho =$-0.608, p-value<0.001). The decline in SampEn becomes more prominent beyond 37 WoG, suggesting a reduction in the randomness of uterine electrical activity as labour approaches. Multiple gestation showed a large dispersion in SampEn, obtaining a near-significant negative correlation with GA ( $\rho =$-0.179, p-value= 0.054).

**FIGURE 3.**
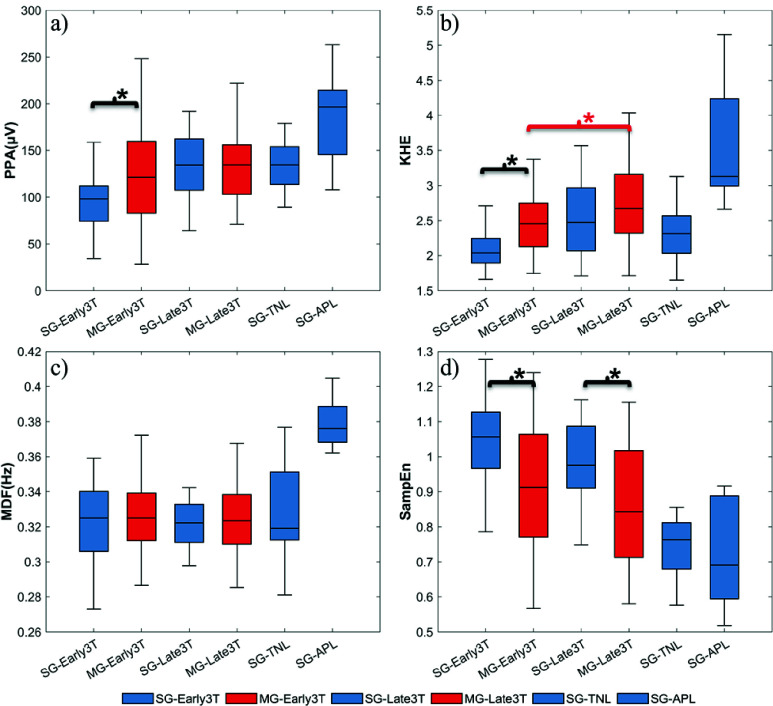
Comparative analysis of EHG feature across gestational stages and pregnancy types (SG vs. MG). Significance levels: $\ast $p <0.05.

To further quantify the changes in EHG features across different gestational stages, Fig. 3 presents boxplots of four key EHG parameters for SG and MG pregnancies. Table 4 provides statistical comparisons across different stages in SG pregnancies.

**TABLE 4:** Statistical Comparisons of EHG Features Across Gestational Stages in Singleton Pregnancies

SG		Late3T	TNL	APL
PPA	Early3T	<0.001	<0.001	<0.001
	Late3T	–	0.807	0.013
	TNL		–	0.016
KHE	Early3T	<0.001	0.010	<0.001
	Late3T	–	0.099	0.007
	TNL		–	0.002
MDF	Early3T	0.409	0.709	<0.001
	Late3T	–	0.457	<0.001
	TNL		–	<0.001
SampEn	Early3T	0.008	<0.001	<0.001
	Late3T	–	<0.001	<0.001
	TNL		–	0.664

At Early3T, MG pregnancies showed significantly higher PPA, KHE values and significantly lower SampEn values than SG pregnancies, suggesting a higher number of recruited uterine muscle cells and a more coordinated electrical activity (see Fig. 3). However, as pregnancy progresses, these differences gradually decrease, and only the SampEn parameter obtained statistically significant differences between SG and MG at the Late3T stage. No statistically significant differences were found in the MDF feature between the SG and MG groups at either the Early3T or in the Late3T stages (p >0.05).

In SG pregnancies, PPA, KHE, and SampEn showed significant differences (p <0.05) between Early3T and each stage of the groups during the third trimester (Table 4). APL showed significant differences with the Early3T and Late3T groups for the four EHG parameters (p <0.05), suggesting that EHG undergoes significant changes during labour, while MG pregnancies showed no significant changes between Early3T and Late3T, except for KHE.

## IV. Discussion

### A. Dynamic Evolution of EHG Features in SG

This study provides the first systematic characterization of the dynamic electrophysiological evolution of EHG signal features in singleton pregnancies, spanning from slightly before the third trimester of pregnancy (>26 WoG) to the active phase of labour. The overall trends offer new insights into common EHG signal change patterns during late pregnancy and provide valuable reference data for future studies on the temporal dynamics of EHG characteristics across different gestational stages.

Our results demonstrate a progressive increase in amplitude-related EHG parameters, such as the PPA, as gestation advances. The gradual elevation in signal amplitude may be associated with the progressive depolarization of the resting membrane potential, shifting from approximately −70 mV in early pregnancy to around −50 mV during the active phase of labour, together with the increased size and number of myocytes involved in the contractions as pregnancy progresses [41], [42].

The parameters KHE and MDF remained relatively stable until a few days before labour onset, after which they exhibited a marked increase. This pattern is likely driven by hormonal regulatory mechanisms in late pregnancy. Progesterone, a key hormone responsible for maintaining uterine quiescence, progressively increases throughout pregnancy, reaching peak levels in the third trimester and remaining high until delivery [43], [44]. In parallel, oxytocin—critical for initiating and sustaining uterine contractility—also rises gradually over the course of gestation [45]. However, the myometrium remains relatively unresponsive during most of pregnancy due to low oxytocin receptor (OXTR) expression [46]. In the final days before delivery, a rise in estrogen levelsleads to a sharp upregulation of OXTR in the myometrial tissue, sensitizing the uterus to oxytocin and enabling the onset of the strong, coordinated contractions that initiate labour [47].

In addition, elevated levels of estrogen and oxytocin in late gestation are known to upregulate the expression of gap junction proteins such as connexin-43, facilitating the formation of gap junctions [48]. This enhances cell-to-cell coupling and improves the synchronicity and propagation of electrical signals. The increased myometrial connectivity consequently reduces signal complexity and randomness, which is reflected in the gradual decrease in SampEn observed throughout gestation in the present study. This finding agrees with Fele-Zorz et al., who proposed SampEn as a reliable biomarker for preterm birth prediction [12]. In comparison with the findings reported by Vrhovec et al. [16], our results align with a continuously decreasing trend obtained from the uterine corpus.

In short, the present findings suggest that, in SG, the uterus maintains a balanced homeostatic state—both hormonally and electrophysiologically—that prevents premature activation of labor pathways and enables a gradual, coordinated adaptation of uterine excitability and contractility throughout gestation, consistent with normal physiological progression [49].

### B. Differences in EHG Features Between Singleton and Multiple Pregnancies

Consistent with the findings reported by Diaz Martínez et al., the present study identified significant differences in EHG signal parameters between SG and MG pregnancies, suggesting that MG may undergo a more accelerated and distinct biophysical progression toward labour [20]. Compared to SG, MG imposes greater mechanical stretch and uterine overdistension, triggering a series of electrophysiological adaptations.

As indicated by prior research, in twin pregnancies, fetal growth lags behind that of singleton pregnancies before 20 WoG, followed by a catch-up growth phase lasting until approximately 24 WoG, after which the growth rate slows down [50]. The mechanical stretching imposed on the uterus in early third trimester is therefore significantly higher in MG than in SG, potentially leading to earlier uterine activation in MG pregnancies, a finding consistent with the results of our study, which demonstrated that the disparities in EHG characteristics between SG and MG were more pronounced during the early third trimester.

Excessive mechanical stretch has been shown to activate various inflammatory signalling pathways, such as those mediated by IL-6 and TNF- $\alpha $, and to upregulate the expression of contraction-associated genes [51]. Uterine stretch also influences key ion channels, such as BKCa and LTCCs, leading to elevated intracellular calcium and increased myometrial excitability [52]. It also promotes collagen synthesis and strengthens myocyte–extracellular matrix adhesion, potentially contributing to heightened uterine activity earlier in gestation in MG pregnancies [53]. These mechanisms may underlie the significant differences in EHG signal characteristics found between MG and SG during the early third trimester, suggesting that MG pregnancies may enter into a labour-prepared state earlier than SG pregnancies and increase the risk of preterm birth.

Compared to SG, MG exhibited a slower progression toward labor, with no significant changes from early to late third trimester except for KHE. This contrasts with the SG group, where EHG features demonstrated significant variations between the early third trimester and the later stages of pregnancy. The sustained mechanical loading may elicit earlier or more persistent adaptive responses in the myometrium, resulting in attenuated changes in EHG features during late gestation, thus possibly leading to reduced differences across gestational stages in MG pregnancies.

Prolonged mechanical stretching of the uterus has been shown to suppress contractility through multiple mechanisms, including upregulation of mechanosensitive potassium channels such as TREK-1, which contribute to membrane hyperpolarization and reduced myometrial excitability [54], [55]. Additionally, the stretch-induced expression of matrix metalloproteinases (MMP-2 and MMP-9) further promotes uterine relaxation, suggesting a physiological safeguard against overdistension and preterm labour [56].

Also, chronic inflammation alters calcium channel expression and ion influx, impairing action potential generation [57], potentially explaining the downward MDF trend observed in MG. This finding suggests that MG is characterized by early activation in Early3T, followed by slower progression in Late3T, reflecting impaired regulatory mechanisms due to disrupted homeostatic balance.

From a translational perspective, these electrophysiological differences highlight the potential of EHG analysis as a noninvasive clinical tool. Recognizing and understanding these differences is essential for the development of tailored preterm birth prediction models and individualized intervention strategies for multiple gestations.

### C. Limitations and Future Lines of Research

This study used an extensive database for analysis and yielded encouraging results; however, certain limitations remain. The segmentation of electrophysiological segments in the EHG signal currently depends on expert visual identification, which is time-consuming and costly, hindering the integration of EHG technology into clinical practice. With the advance of automated monitoring systems for detecting uterine contractions and physiological signals [31], future research could focus on automating the identification and extraction of EHG signals, thus enhancing the portability of EHG technology for clinical use.

Secondly, while our analysis did not differentiate between preterm and full-term births, this is not a major limitation given that the vast majority of SG cases (53 out of 56) were full-term. Data from MG during TNL and APL stages would offer valuable insight into the electrophysiological changes preceding labour, although such data remain difficult to obtain due to the high PTB incidence in MG plus the logistical challenges during active labour monitoring.

Thirdly, we did not assess the sensitivity of EHG parameters to different electrode arrangements, which is of significant scientific and clinical interest. Such an analysis would require testing multiple configurations, substantially increasing study complexity and potentially reducing patient acceptability due to over-monitoring or the need for a randomized design with a large dataset. In this regard, future work may use computational modeling [58]—which has previously been shown to reproduce uterine myoelectric propagation—to explore optimal electrode spacing and promote standardization.

Despite its limitations, this study significantly enhances the understanding of the physiological characteristics of uterine EHG in MG, offering valuable technical support for further insights into preterm birth (PTB) in MG in clinical practice.

## V. Conclusion

Our results showed that SG pregnancies undergo more pronounced changes in EHG parameters throughout late pregnancy, particularly near labour onset, whereas MG pregnancies demonstrate higher uterine excitability and contraction regularity before 32 WoG. These findings suggest that MG pregnancies may enter a labour-prepared state earlier than SG pregnancies, increasing the risk of preterm birth. As pregnancy progresses, uterine electrical activity in SG pregnancies intensifies to a greater extent than in MG, reducing the differences between the two groups. The differences in the progression of uterine electrophysiological status between SG and MG during the third trimester of pregnancy are of paramount importance for monitoring pregnancy and the development of preterm birth prediction systems to improve preterm birth risk assessment and promote intervention strategies, particularly in the early third trimester.
